# MMFuncPhos: A Multi‐Modal Learning Framework for Identifying Functional Phosphorylation Sites and Their Regulatory Types

**DOI:** 10.1002/advs.202410981

**Published:** 2025-01-13

**Authors:** Juan Xie, Ruihan Dong, Jintao Zhu, Haoyu Lin, Shiwei Wang, Luhua Lai

**Affiliations:** ^1^ Center for Quantitative Biology Academy for Advanced Interdisciplinary Studies Peking University Beijing 100871 China; ^2^ PTN Graduate Program Academy for Advanced Interdisciplinary Studies Peking University Beijing 100871 China; ^3^ Peking‐Tsinghua Center for Life Sciences at BNLMS College of Chemistry and Molecular Engineering Peking University Beijing 100871 China; ^4^ Peking University Chengdu Academy for Advanced Interdisciplinary Biotechnologies Chengdu Sichuan 610213 China; ^5^ Research Unit of Drug Design Method Chinese Academy of Medical Sciences (2021RU014) Beijing 100871 China

**Keywords:** drug discovery, enzyme engineering, functional phosphorylation sites, multi‐modal deep learning framework, phosphorylation regulation types, precision medicine, transfer learning

## Abstract

Protein phosphorylation plays a crucial role in regulating a wide range of biological processes, and its dysregulation is strongly linked to various diseases. While many phosphorylation sites have been identified so far, their functionality and regulatory effects are largely unknown. Here, a deep learning model MMFuncPhos, based on a multi‐modal deep learning framework, is developed to predict functional phosphorylation sites. MMFuncPhos outperforms existing functional phosphorylation site prediction approaches. EFuncType is further developed based on transfer learning to predict whether phosphorylation of a residue upregulates or downregulates enzyme activity for the first time. The functional phosphorylation sites predicted by MMFuncPhos and the regulatory types predicted by EFuncType align with experimental findings from several newly reported protein phosphorylation studies. The study contributes to the understanding of the functional regulatory mechanism of phosphorylation and provides valuable tools for precision medicine, enzyme engineering, and drug discovery. For user convenience, these two prediction models are integrated into a web server which can be accessed at http://pkumdl.cn:8000/mmfuncphos.

## Graphical abstract

The functions of most phosphorylation sites remain unknown, which is the “bottleneck” of phosphorylation modification studies. Here, a state‐of‐the‐art method, MMFuncPhos, is developed to predict functional phosphorylation sites based on a multi‐modal deep learning framework. EfuncType is further developed based on transfer learning to predict whether phosphorylation of a residue upregulates or downregulates enzyme activity for the first time.

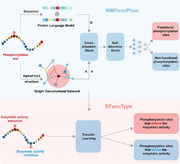

## 1 Introduction

Post‐translational modifications (PTMs) are essential biochemical reactions that covalently regulate protein conformation, activity, and stability, playing key roles in a wide range of biological processes.^[^ ^1^, ^2^ ^]^ Dysregulation of PTM is closely related to various human diseases, such as cancers, neurodegenerative and cardiovascular disorders as well as diabetes.^[^ ^3^, ^4^ ^]^ Phosphorylation is one of the most pervasive and important PTMs, intimately related to almost every cellular process, and has the largest number of disease associations.^[^ ^5^, ^6^, ^7^ ^]^ Previous studies suggest that up to 75% of the yeast and human proteomes are phosphorylated, and more than 150 000 non‐redundant human phosphorylation sites have been discovered, primarily through mass spectrometry.^[^ ^8^, ^9^ ^]^ However, currently known functional phosphorylation sites comprise less than 5% of the total identified phosphorylation sites, and functional research is the “bottleneck” problem in the field of phosphorylation modification studies.^[^ ^10^ ^]^ Evolutionary studies indicated that 35–65% of phosphorylation sites are functional, suggesting a large proportion of phosphorylation sites may not be functional.^[^ ^11^, ^12^ ^]^ Since experimental methods are time‐consuming and have limitations in comprehensively characterizing the functions of PTM sites,^[^ ^13^ ^]^ there is an urgent need to develop computational methods to determine whether phosphorylation sites are functional and to identify their specific functions.

Compared to computational methods for predicting phosphorylation sites, such as DeepPhos,^[^ ^14^ ^]^ PhosIDN,^[^ ^15^ ^]^ and DeepMPSF,^[^ ^16^ ^]^ computational methods for predicting functional phosphorylation sites are less developed. Su et al. developed CruxPTM, an integrated platform for analyzing the structural characteristics and biological functions of PTM sites.^[^ ^17^ ^]^ Ochoa et al. used the gradient‐boosting machine model to combine 59 features, including proteomic, structural, evolutionary, or regulatory relevance, into a single functional score to predict functional phosphorylation sites.^[^ ^18^ ^]^ Li et al. developed the machine learning‐based method named iFPS, which integrates six frequently used sequence and structural features to evaluate the functionality of the candidate phosphorylation sites in *Caenorhabditis elegans*.^[^ ^19^ ^]^ Zhu et al. developed two deep learning models, cDL‐PAU and cDL‐FuncPhos, to predict whether a site is a phosphorylation, acetylation, or ubiquitination site and to predict whether a phosphorylation site has biological function.^[^ ^20^ ^]^ Liang et al. proposed FuncPhos‐SEQ, a deep neural network model for predicting functional phosphorylation sites, which incorporates motif information of phosphorylation sites and protein‐protein interaction network features.^[^ ^21^ ^]^ However, the accuracy of these methods in predicting functional phosphorylation sites remains to be improved, and there is currently no effective computational method that can predict the regulatory types of phosphorylation sites on enzyme activity.

Phosphorylation plays a crucial role in regulating enzyme activity.^[^ ^22^ ^]^ Previous researches have shown that phosphorylation at certain sites can either inhibit or enhance enzyme activity. For example, Zhou et al. investigated the role of tyrosine phosphorylation of Janus kinase JAK3 in regulating its kinase activity by analyzing mutations of tyrosine residues within the putative activation loop of the kinase domain. They found that JAK3 was autophosphorylated at multiple sites, including Y980 and Y981, and that Y980 positively regulated JAK3 kinase activity, whereas Y981 negatively regulated JAK3 kinase activity.^[^ ^23^ ^]^ Like JAK3, protein tyrosine kinase ZAP‐70 has repeated tyrosine residues Y492 and Y493 in the putative activation loop of the kinase domain. Previous studies have found that phosphorylation of Y492 inhibited kinase activity and antigen receptor function, while phosphorylation of Y493 enhanced kinase activity, which is required for lymphocyte antigen receptor function.^[^ ^24^, ^25^, ^26^ ^]^ As can be seen, although the phosphorylation sites are in the same protein (sometimes even adjacent), the functional regulatory types after phosphorylation are completely different. Whether phosphorylation at a site enhances or inhibits enzyme activity can only be revealed after conducting time‐consuming experiments. Information on the regulatory types of phosphorylation sites will expedite related research for desired function regulation. Unfortunately, currently no effective computational method can a priori predict whether phosphorylation of a certain residue will upregulate or downregulate enzyme activity.

Multi‐modal methods, which can integrate data from multiple modalities, exhibit remarkable generalization capabilities for new categories and tasks, and have been widely used in various areas such as the enzyme active site prediction and the molecule representation learning.^[^ ^27^, ^28^ ^]^ Currently, some protein sequences annotated with functional phosphorylation sites are easily accessible, but crystal structures containing functional phosphorylation sites are very scarce.^[^ ^29^ ^]^ Integrating structural information presents a highly intuitive way to drive the structure‐based functional site prediction and description of the local structural environment of sites. Fortunately, with the rapid advancement of protein structure prediction, it is able to access the predicted protein structures of over 214 million protein sequences in the AlphaFold Protein Structure Database (AlphaFold DB).^[^ ^30^ ^]^ In addition, there is an emerging trend to leverage pre‐trained protein language models to extract protein representations that contain rich evolutionary context.^[^ ^31^, ^32^ ^]^ We believe that the sequence representations from protein language models and the structural information can complement each other effectively, and functional phosphorylation site prediction can greatly benefit from integrating both by the multi‐modal approach.

**Figure 1:**
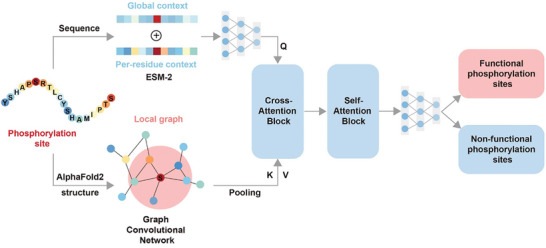
Multi‐modal deep learning model MMFuncPhos for predicting functional phosphorylation sites. The model mainly consists of two parts. One is based on the protein language model ESM‐2 to extract the sequence evolutionary information, and the other is based on the GCN to encode the local structure graph of phosphorylation sites. Cross‐attention block is used to merge the representations of the local structure graph and sequence evolutionary information, and its output will pass through a self‐attention block. Finally, a 3‐layer full‐connected neural network is used to predict functional phosphorylation sites.

In the present study, we proposed MMFuncPhos, a deep learning method designed for efficiently predicting functional phosphorylation sites based on a multi‐modal deep learning framework. MMFuncPhos employed the protein language model ESM‐2 to extract both the global and residue‐level evolutionary information of phosphorylation sites and used the graph convolutional network (GCN) to extract the local structure graph of phosphorylation sites. These features were fused using a cross‐attention block to determine whether a phosphorylation site is functional (**Figure** **1**). We adopted the transfer learning strategy to further develop EFuncType, a method for predicting the regulatory types of phosphorylation sites on enzyme activity (Figure 5). EFuncType is the first method to achieve a priori prediction of whether phosphorylation of certain sites enhance or inhibit enzyme activity. MMFuncPhos and EFuncType successfully identified the functional phosphorylation sites and regulatory types in several newly reported proteins, including SERCA2, Src, and NADK. To facilitate user access, we developed an integrated web server that contains both MMFuncPhos and EFuncType. These tools are valuable for understanding the functional regulatory mechanism of phosphorylation and provide useful resources for precision medicine, enzyme engineering, and drug discovery.

## 2 Results and Discussion

### 2.1 Statistical Analysis of the Features Underlying Functional Phosphorylation Sites

**Figure 2:**
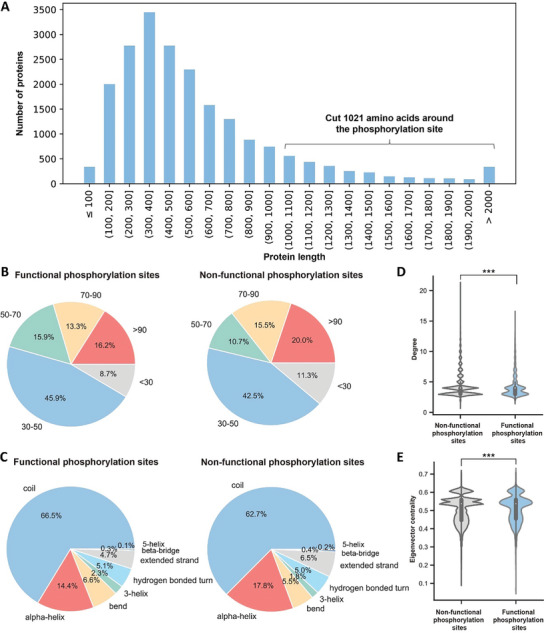
Statistical analysis of features of phosphorylation sites in the data set. A) Distribution of the protein sequence length. B) Distribution of the pLDDT of the functional and non‐functional phosphorylation sites. C) Distribution of the secondary structures of the functional and non‐functional phosphorylation sites. D,E) Distribution of the degree and eigenvector centrality of the functional and non‐functional phosphorylation sites. Two‐tailed unpaired *t*‐test was used for statistical analysis. ^***^ *p* < 0.001.

We collected the information of phosphorylation sites from the PhosphoSitePlus (PSP) database (version 22‐11‐18).^[^ ^8^, ^29^ ^]^ In PSP, there are 378636 phosphorylation sites in total, among which 15317 sites are annotated with downstream regulatory functions, and 2373 disease‐associated sites. We regarded these regulatory sites and disease‐associated phosphorylation sites in PSP as functional sites, and those without annotations as non‐functional sites. Finally, we got 7245 functional phosphorylation sites and 179610 non‐functional phosphorylation sites. For all proteins in the data set that contain these phosphorylation sites, the distribution of protein sequence length shows that the protein lengths in the data set range from 51 to 2753 residues, with the majority of proteins containing 300–400 residues and a small proportion of proteins being longer than 1000 residues (**Figure** **2**A).

Since the AlphaFold2 (AF2) predicted protein structures were used here,^[^ ^33^, ^34^ ^]^ we first analyzed the distributions of the per residue local distance difference test (pLDDT) of these functional and non‐functional phosphorylation sites. Figure 2B shows that the functional phosphorylation sites are more frequently located in low (50 < pLDDT < 70) or very low (pLDDT < 50) confidence regions than non‐functional phosphorylation sites (70.5% vs 64.5%).

We also analyzed the distribution of functional and non‐functional phosphorylation sites on protein secondary structures. It can be seen that known functional phosphorylation sites prefer to be located on coils (66.5%) compared to alpha‐helix and other secondary structures, and functional phosphorylation sites are also more likely located on coils than non‐functional phosphorylation sites (Figure 2C). We then compared the degree and eigenvector centrality distributions of the functional and non‐functional phosphorylation sites. The results showed that there were significant differences in the degree and eigenvector centrality distributions of the functional and non‐functional phosphorylation sites, with *p* values of 2.25e‐05 and 1.13e‐06, respectively (Figure 2D,E). The lower degree of functional phosphorylation sites may be due to many of them being located in the coil region, while the higher eigenvector centrality of functional phosphorylation sites indicates that they play a critical role in the flow of phosphorylation regulatory information.

### 2.2 Performance Evaluation of the Functional Phosphorylation Site Prediction Models

**Figure 3:**
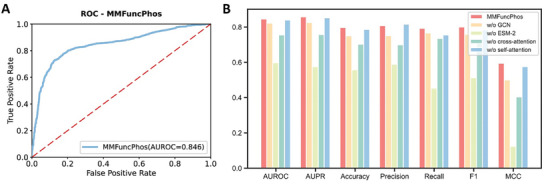
Performance of the MMFuncPhos model and other models in the ablation experiments. A) The AUROC of MMFuncPhos model in predicting functional phosphorylation sites. B) Results of ablation studies. MMFuncPhos is the final model, and the remaining four models are the models of ablation experiments. w/o GCN: GCN module was removed from the MMFuncPhos; w/o ESM‐2: ESM‐2 module was removed from the MMFuncPhos; w/o cross‐attention: cross‐attention block was removed from the MMFuncPhos; w/o self‐attention: self‐attention block was removed from the MMFuncPhos.

To predict functional phosphorylation sites, we constructed a multi‐modal deep learning model MMFuncPhos (Figure 1; see Experimental section for details). The input of the model consists of two parts. In the first part, the protein sequence is passed into the protein language model ESM‐2 to generate global and residue‐level evolutionary information for each phosphorylation site, with a 2560‐dimensional vector representation of each residue. In the second part, the protein structure is passed into the GCN to generate a local structure graph centered on each phosphorylation site. Each node in the local graph has a 35‐dimensional feature vector. In GCN, a pooling layer was used to compact a batch of spatial subgraphs for more semantic features. Cross‐attention and self‐attention modules were used to fuse the two parts of input information. Finally, a fully connected neural network output a probability value indicating the probability that a site is a functional phosphorylation site. Five‐fold cross‐validation was used to optimize parameters and obtain the model with the best results. We considered different ratios of positive to negative samples, such as 1:1, 1:2, and 1:3, and each experiment was repeated three times. We found that the overall evaluation metrics of the test set were the best when the ratio of positive to negative samples was 1:1, so we finally chose 1:1 (Figure S1, Supporting Information). We also evaluated the effects of different parameters on model performance, including the distance radius of structural graph, batch size, learning rate, dropout rate, and the number of GCN layers. The detailed comparison results can be found in Table S4 (Supporting Information). Finally, we selected the optimal set of parameters as the parameters of the final model. On the test set, the final model MMFuncPhos achieved AUROC of 0.846, AUPR of 0.858, Accuracy of 0.800, Precision of 0.808, Recall of 0.793, F1 of 0.801, and MCC of 0.595 (**Figure** **3**A).

To further investigate the effects of each module in our MMFuncPhos architecture, we conducted ablation study. Our final model contains four main modules, namely GCN module, ESM‐2 module, cross‐attention module, and self‐attention module. We successively removed these four modules from the final model to obtain four different models: 1) w/o GCN: GCN module was removed from the MMFuncPhos; 2) w/o ESM‐2: ESM‐2 module was removed from the MMFuncPhos; 3) w/o cross‐attention: cross‐attention block was removed from the MMFuncPhos; 4) w/o self‐attention: self‐attention block was removed from the MMFuncPhos; and then compared the performance of these four models with the full model MMFuncPhos (Figure 3B and Table S5, Supporting Information). It can be seen that our full model surpassed the other four models in all the evaluation metrics except precision, indicating that all these modules are useful in improving the performance of the full model. Moreover, it is worth noting that sequence evolutionary features from the ESM‐2 module are the most important for improving the predictive performance of the model. At the same time, the cross‐attention module is also very important. When we used the direct concatenation of both the GCN and ESM‐2 outputs to replace the cross‐attention merging, the prediction results of the model became much worse. At the same time, the GCN module and self‐attention module also helped to improve the performance of the full model. Overall, these results highlight the importance of sequence evolutionary information and cross‐attention module in characterizing functional phosphorylation sites.

Since we adopted structures predicted by AF2, we also explored the impact of pLDDT scores on the predictions of the model in the following ways. First, we calculated the correlation between the pLDDT scores of sites in the test set and the probabilities predicted by our model. The results showed that the Spearman correlation coefficient between the pLDDT scores and the predicted probabilities was 0.011, indicating that the correlation between the prediction results of our model and the pLDDT scores was weak. Second, we divided the sites in the test set into different groups according to the pLDDT scores, namely pLDDT > 90, 70 < pLDDT < 90, 50 < pLDDT < 70, 30 < pLDDT < 50, and pLDDT < 30, and then make predictions for each group. The results showed that when pLDDT was less than 70, the prediction results (Recall) of our model improved as the pLDDT score increased, but when pLDDT was greater than 70, the prediction results decreased slightly, that is, the prediction results in different groups did not completely improve as the pLDDT score increased (Figure S2, Supporting Information). Third, we removed pLDDT scores from the model features, which resulted in a decrease of ≈0.02 in accuracy, precision, recall, F1, and MCC, indicating that the pLDDT feature is useful for improving the performance of our model. Overall, these results suggest that there is a correlation between the predictions of our model and the pLDDT scores, but the correlation is weak. Our model has a good generalization and can not only predict functional phosphorylation sites in structured regions well, but also predict functional phosphorylation sites in disordered or loop regions effectively.

### 2.3 Comparison with Other Functional Phosphorylation Site Prediction Methods

**Figure 4:**
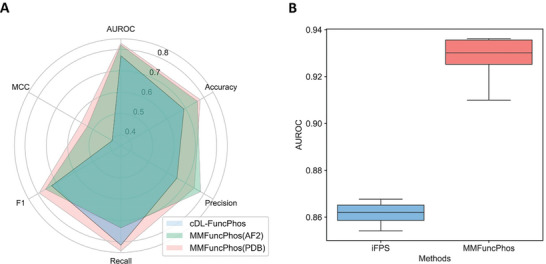
Results of comparison with two representative methods for predicting functional phosphorylation sites. A) Comparison with cDL‐FuncPhos. MMFuncPhos(AF2) represents MMFuncPhos using AF2 structures and MMFuncPhos(PDB) represents MMFuncPhos using PDB crystal structures. B) Comparison with iFPS.

To further evaluate our MMFuncPhos method, we subsequently compared it with representative functional phosphorylation site prediction methods cDL‐FuncPhos and iFPS.^[^ ^19^, ^20^ ^]^ cDL‐FuncPhos exploited abundant properties from sequences, structures, and dynamics as input features to recognize functional phosphorylation sites. To do a fair comparison, we retrained and tested our model on the training and test sets of cDL‐FuncPhos using PDB crystal structures as cDL‐FuncPhos did. The test results showed that the AUROC, Accuracy, Precision, Recall, F1, and MCC of our method were all higher than those of cDL‐FuncPhos (**Figure** **4**A). We further evaluated our model performance on the AF2 structures, and the test results showed that the AUROC, Accuracy, Precision, F1 and MCC of our method were higher than those of cDL‐FuncPhos (Figure 4A), especially the precision, which is 0.128 higher than that of cDL‐FuncPhos and closely linked to the success rate of wet‐lab experiments, demonstrating that our model based on the AF2 structures can also predict functional phosphorylation sites well, making it highly practical. In practical applications, many proteins containing functional phosphorylation sites do not have high‐quality crystal structures and the structures predicted by AF2 are easily accessible. Therefore, a model robust to AF2 structures is what scientists would like to use. Overall, MMFuncPhos shows state‐of‐the‐art performance and robustness either on crystal structures or AF2 structures, making it useful in practice.

iFPS focused on *C.elegans*, with 121 validated functional sites and 10698 non‐functional sites, and mainly integrated six frequently used sequence and structural features to evaluate the functionality of the candidate phosphorylation sites. In order to compare to iFPS, we divided the positive set of 121 functional phosphorylation sites and randomly selected 10 negative sets of non‐functional sites into training and independent test sets in a 4:1 ratio, and performed a similar cross‐validation. MMFuncPhos excels iFPS with an average AUROC score of 0.928 (± 0.023), which is higher than that of iFPS (0.862 (± 0.009)) (Figure 4B). Overall, our model outperformed other functional phosphorylation site prediction methods.

### 2.4 Regulatory Type Prediction of Phosphorylation on Enzyme Activity

Phosphorylation plays a crucial role in regulating enzyme activity. Previous researches have shown that phosphorylation at certain sites can either inhibit or enhance enzyme activity. To predict how different phosphorylation sites affect enzyme activity, we collected data on phosphorylation sites that have inhibitory or enhancing effects (Table S2, Supporting Information). In total, we obtained 743 phosphorylation sites that enhance enzyme activity and 300 phosphorylation sites that inhibit enzyme activity. Given the small size of this data set, we explored several training strategies for small‐scale learning. Initially, we trained a model directly on the collected data, which resulted in an AUROC value of 0.694.

We then attempted a multi‐task training approach, training the enzyme activity data set alongside the larger functional phosphorylation site data set at the same time with a joint loss function (the sum of two binary cross‐entropy losses). However, as the larger size of the functional data set dominated, the AUROC value for enzyme regulatory type prediction only improved to 0.732.

**Figure 5:**
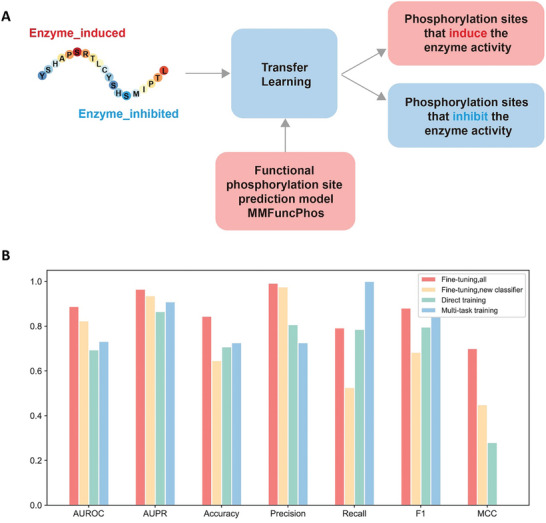
A transfer learning‐based model EFuncType for predicting the regulatory types of phosphorylation on enzyme activity. A) The frame of the EFuncType model. B) Performance of the EFuncType (Fine‐tuning, all) and other models.

Finally, we employed a transfer learning strategy. We tested two approaches: the first was to transfer parameters of all embedding layers except the classifier from the best model of functional phosphorylation site prediction as the initial parameters for the regulatory type prediction model, and the second was to transfer the entire parameters from the best model of the functional phosphorylation site prediction as the initial parameters for the model here. Then we used the enzyme activity regulatory site data to fine‐tune a new regulatory type prediction model that can predict the regulatory type of phosphorylation on enzyme activity (**Figure** **5**A). The results illustrated that transfer learning was effective, with the best performance achieved by fine‐tuning the entire trained model. The final model, EFuncType, achieved an AUROC of 0.888 and an accuracy of 0.844 (Figure 5B and Table S6, Supporting Information).

### 2.5 Effective Prediction of Functional Phosphorylation Sites and Regulatory Types by MMFuncPhos and EFuncType on Newly Reported Examples

To further evaluate the effectiveness of our method, we collected data on functional phosphorylation sites newly added to the PSP from November 18, 2022, to October 18, 2023, as a new independent test set, which contained 475 newly added regulatory sites, 96 disease‐associated sites, 22 sites labeled as “enzymatic activity, inhibited” and 39 sites labeled as “enzymatic activity, induced”. For this new test set, MMFuncPhos correctly predicted 72.9% of the disease‐associated sites and 64.4% of the regulatory sites. EFuncType achieved a recall of 0.769 and a precision of 0.698.

**Figure 6:**
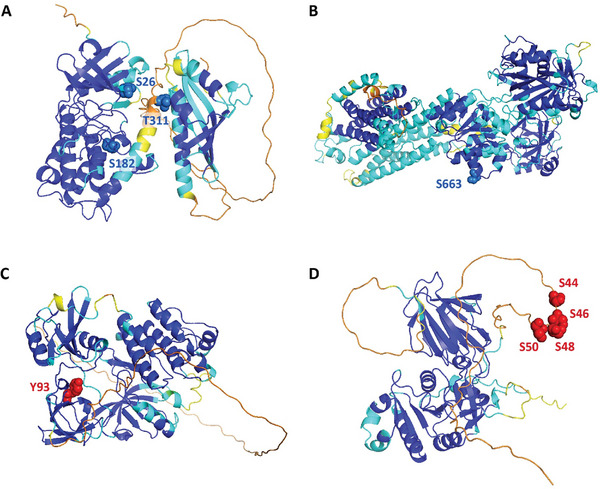
MMFuncPhos predicted functional phosphorylation sites and EFuncType predicted regulatory types of phosphorylation sites in CaMKIIβ (A), SERCA2 (B), Src (C), and NADK (D). The true functional phosphorylation sites predicted by MMFuncPhos are shown as red or blue spheres, where the red spheres represent experimentally discovered sites that can enhance enzyme activity, and the blue spheres represent sites that can inhibit enzyme activity. The phosphorylation sites further predicted by EFuncType that truly enhance enzyme activity are marked with red labels, the sites predicted by EFuncType that truly inhibit enzyme activity are marked with blue labels. The structures of these proteins were shown as cartoon and colored according to pLDDT.

One example is Ca^2+^/calmodulin‐dependent protein kinase II (CaMKII)β, a sleep‐promoting kinase that plays an important role in mammalian sleep regulation.^[^ ^35^ ^]^ Phosphorylation states of CaMKIIβ can control sleep induction and maintenance.^[^ ^36^ ^]^ Previous studies identified Ser26, Ser182, and Thr311 as functional phosphorylation sites that inhibit CaMKIIβ kinase activity, potentially eliminating its sleep‐promoting effect.^[^ ^36^ ^]^ These sites are highly conserved among different species. Using MMFuncPhos, we correctly predicted these three sites as functional phosphorylation sites with probabilities of 0.998, 0.906, and 0.998, respectively. We further used EFuncType to predict the regulatory types of these three sites. EFuncType was able to correctly predict them as inhibiting enzyme activity with probabilities of 0.068, 0.375, and 0.441, respectively (**Figure** **6**A). These results further demonstrate the effectiveness of both MMFuncPhos and EFuncType.

To further validate the effectiveness of our approaches, we also tested them on three recently published protein phosphorylation examples outside the data set and the independent test set. The first protein, sarcoplasmic/endoplasmic reticulum calcium ATPase 2 (SERCA2), plays a key role in coordinating Ca^2+^ handling in cardiac muscle and is a promising target for treating contractile dysfunction following ischemia‐reperfusion.^[^ ^37^ ^]^ A recent study highlighted that the phosphorylation level of SERCA2 at Ser663 critically regulates Ca^2+^ homeostasis and cell fate in the heart. Phosphorylation at this site negatively affects SERCA2 activity, leading to reduced sarco/endoplasmic reticulum Ca^2+^ content and a consequent Ca^2+^ overload in both the cytosol and mitochondria, making cells sensitive to death. Preventing phosphorylation at Ser663 can offer in vivo protection against reperfusion injury.^[^ ^38^ ^]^ MMFuncPhos correctly predicted Ser663 as a functional phosphorylation site with a probability of 0.939 and EFuncType successfully predicted that phosphorylation of Ser663 inhibits SERCA2 activity with a probability of 0.242 (Figure 6B).

The second protein, proto‐oncogene tyrosine‐protein kinase Src (Src), is the founding member of the Src family non‐receptor tyrosine kinases. It plays a crucial role in various signaling and metabolic pathways such as cell differentiation and proliferation.^[^ ^39^ ^]^ Aberrant activation of Src leads to cellular transformation and has been found to be associated with various cancers such as gastric cancer, lung cancer, and pancreatic cancer.^[^ ^40^, ^41^ ^]^ The activity of Src kinase is often allosterically regulated by numerous cellular signals. A recent study found that phosphorylation of Tyr93 allosterically increased Src kinase activity.^[^ ^42^ ^]^ MMFuncPhos correctly predicted Tyr93 as functional phosphorylation site with a probability of 0.999. At the same time, EFuncType successfully predicted that phosphorylation of Tyr93 can increase Src kinase activity with a probability of 0.659 (Figure 6C).

The third protein, NAD kinase (NADK) catalyzes the synthesis of NADP^+^ from NAD^+^ and is crucial for regulating the intracellular NADP^+^/NADPH content to adapt to the complex intracellular and extracellular environment.^[^ ^43^ ^]^ Previous studies identified Ser44, Ser46, Ser48, and Ser50 as functional phosphorylation sites that positively regulate its enzyme activity.^[^ ^21^, ^44^, ^45^ ^]^ MMFuncPhos successfully predicted Ser48, Ser46, Ser44, and Ser50 as functional phosphorylation sites with probabilities of these four sites were all very high, with probabilities of 0.999, 0.999, 0.998, and 0.994, respectively. Furthermore, EFuncType successfully predicted that phosphorylation of these sites enhance enzyme activity with probabilities of 0.538, 0.559, 0.558, and 0.618, respectively (Figure 6D).

In these three newly reported protein systems that contain phosphorylation sites that either enhance or inhibit enzyme activity, MMFuncPhos correctly predicts them as functional phosphorylation sites and EFuncType successfully predicts their regulatory types, demonstrating the efficient prediction capabilities of MMFuncPhos and EFuncType.

### 2.6 MMFuncPhos Web Server

**Figure 7:**
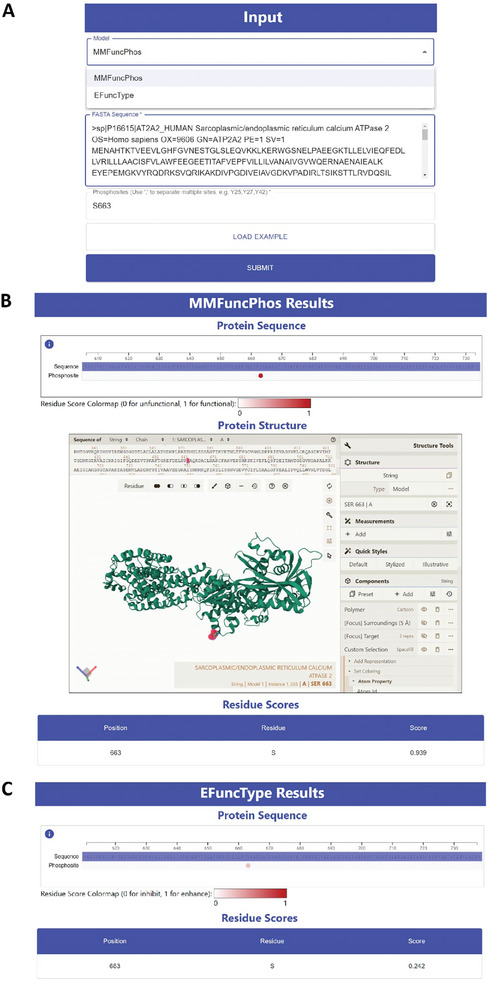
The example input and output of MMFuncPhos web server. A) The input interface of the server. B) The MMFuncPhos result page of the server. The page includes protein sequence, protein structure, and the score of the input phosphorylation site predicted by MMFuncPhos. C) The EFuncType result page of the server.

For user convenience, we developed a comprehensive web server MMFuncPhos for predicting functional phosphorylation sites and their regulatory types, which contains two modules: MMFuncPhos and EFuncType (http://pkumdl.cn:8000/mmfuncphos). Users only need to input the protein sequence and the phosphorylation sites they want to predict (**Figure** **7**A).

Generally speaking, we often choose to incorporate as many features as possible into the model to enhance its predictive performance. However, complex feature processing elevates the difficulty for usage. As for our models, though most AF2 structures are relatively easy to get, modeling some long sequences with AF2 is time‐consuming. For convenient use, we trained plain MLP models with only sequence inputs and ESM‐2 embeddings (Figure S3, see Supporting Information for details). We distilled the AF2 structural subgraph‐related information into the new three‐layer predictors. For the prediction of functional phosphorylation sites, the distilled MLP had an AUC value of 0.842, an AUPR of 0.837, and a recall of 0.780 (Figure S4, Supporting Information), which is close to the performance of the teacher model and better than the ablation model without structural input. For the prediction of the regulatory types of phosphorylation on enzyme activity, the distilled MLP had an AUC value of 0.794, an AUPR of 0.904, and a recall of 0.903 (Figure S5, Supporting Information), which is close to the performance of the teacher model. The MMFuncPhos web application is developed using a front‐end and back‐end separation approach, featuring independent rendering and business logic. Its user interface is implemented using the front‐end JavaScript framework React. Its components adopt Material style UI, sequence visualization utilizes rcsb‐saguaro,^[^ ^46^ ^]^ and molecular visualization utilizes the Molstar toolkit.^[^ ^47^ ^]^ The business logic is based on the back‐end Python framework Flask, providing a RESTful API service for the front end. Upon receiving computational requests from the front‐end, the back‐end invokes our deep learning model, implemented with PyTorch and DGL, to perform inference and return responses.

The results page includes three parts: protein sequence, protein structure, MMFuncPhos results, or EFuncType results. The protein sequence section displays the positions of the predicted phosphorylation sites within the sequence and colors the residues based on the predicted scores. The protein structure section shows the 3D structure of the protein from the AlphaFold DB and the position of the input phosphorylation site in the protein structure. The MMFuncPhos results give the predicted scores for the requested phosphorylation sites. Sites with scores greater than 0.5 will be predicted as functional phosphorylation sites (Figure 7B). The EFuncType results give the scores for the input phosphorylation sites. Phosphorylation of sites with scores greater than 0.5 will be predicted to enhance enzyme activity, while those with scores less than 0.5 will be predicted to inhibit enzyme activity (Figure 7C).

## 3 Conclusion

Protein phosphorylation regulates various biological processes and is closely linked to the development of many human diseases including cancer and neurodegenerative disorders. Although many phosphorylation sites have been identified, the majority remain to be determined in terms of functionality and specific regulatory types. To address these challenges, we first developed a novel method, MMFuncPhos, to predict functional phosphorylation sites based on a multi‐modal deep learning framework. MMFuncPhos outperformed other functional phosphorylation site prediction methods. We further developed EFuncType based on transfer learning to predict the regulatory types of phosphorylation of specific sites on enzyme activity. To the best of our knowledge, EFuncType is the first method that can predict a priori whether phosphorylation of a residue can enhance or inhibit enzyme activity.

MMFuncPhos and EFuncType successfully identified experimentally validated functional phosphorylation sites and their regulatory types in three newly published protein systems including SERCA2, Src, and NADK. For user convenience, we also developed the MMFuncPhos web server. Identifying and understanding functional phosphorylation sites and their regulatory types provides essential information for precision medicine, drug discovery, and enzyme engineering. MMFuncPhos and EFuncType provide powerful tools for phosphorylation studies and the MMFuncPhos web server can be accessed at http://pkumdl.cn:8000/mmfuncphos.

## 4 Experimental Section

### The Functional Phosphorylation Site Data Set

The information of phosphorylation sites was collected from the PSP database (version 22‐11‐18).^[^ ^8^, ^29^ ^]^ In PSP, there were 378636 phosphorylation sites in total, among which 15317 sites were annotated with downstream regulatory functions, and 2373 disease‐associated sites. These regulatory sites and disease‐associated phosphorylation sites in PSP were regarded as functional sites, and those without annotations as non‐functional sites.

Protein sequences containing these phosphorylation sites were downloaded from the UniProt database and filtered by:^[^ ^48^ ^]^ 1) removing the isoform proteins; 2) removing redundancy sequences using cd‐hit with a similarity threshold of 0.8;^[^ ^49^ ^]^ 3) removing proteins for which 3D structures were not found in the AlphaFold DB using UniProt IDs;^[^ ^33^, ^34^ ^]^ 4) ensuring each phosphorylation site can match its protein sequence and the corresponding AF2 structure model, with the same residue on the corresponding sites.

Finally, a total of 7245 functional phosphorylation sites and 179610 non‐functional phosphorylation sites were obtained. Due to the severe imbalance between positive (functional) and negative (non‐functional) sites, the negative sites were under‐sampled at the ratio of 1:1. The training/test sets were randomly split at a ratio of 4:1 approximately (Table S1, Supporting Information) and the training was performed with five‐fold cross‐validation. Since more than hundred thousand non‐functional sites were obtained, three independent negative sets were built.

### The Enzyme Activity Regulatory Site Data Set

To specify the functional regulatory types of phosphorylation sites, PSP provided several functional descriptions such as “activity, induced”, “enzymatic activity, inhibited,” and “protein conformation”, etc. The focus was on the phosphorylation sites in enzymes. According to the annotations from PSP, a total of 690 enzymes with EC numbers were selected from 2372 proteins in the above positive set. There were 2257 functional phosphorylation sites related to these enzymes, 1043 of which were annotated with enzymatic activity. To avoid conflicting labels, the sites with “enzymatic activity, inhibited” were considered as the inhibited class, while the rest sites with just “enzymatic activity, induced” as the induced class. The detailed numbers of each class are recorded in Table S2 (Supporting Information).

### Model Architecture

Previous studies have suggested that the evolutionary and topological structural information matter when predicting the functions of specified sites.^[^ ^20^ ^]^ Inspired by this knowledge, a deep learning‐based framework was designed to merge the global and residue‐level evolutionary information of the modified protein and the local structural characterizations around the phosphorylation site. Multi‐modal inputs for a phosphorylation site were its protein sequence and spatial structure (Figure 1).

### Global and Residue‐Level Evolutionary Context

One part of our multi‐modal deep learning model was a global and residue‐level evolutionary context extractor. To accelerate the embedding of protein sequences, an advanced protein language model ESM, which was pretrained on millions of protein sequences was utilized.^[^ ^31^ ^]^ Unsupervised learning on abundant known protein sequences makes the ESM model capable of exploiting the evolutionary context. The language model regards amino acid letters as input and utilizes attention mechanism similar to BERT with random masking. Here, the latest version of ESM, ESM‐2, was chosen to generate protein sequence embeddings.^[^ ^50^ ^]^ A linear layer was added subsequent to the ESM‐2 module to change the dimensions.

### Local Structure Graph

Another part of our model was a GCN module that encodes the local structure graph of phosphorylation sites. The Python library Graphein was used to read the protein structure and encode them into graphs.^[^ ^51^ ^]^ For each phosphorylation site, it was considered the center of the subgraph and expanded to its neighbor residues within eight angstroms.

The *C*_α_ atom of each residue was defined as the graph node. For node features, several properties of residues were chosen: 1) amino acid residue type, a one‐hot encoded vector; 2) relative solvent accessibility (RSA), calculated using DSSP;^[^ ^52^ ^]^ 3) pLDDT, from AF2 predictions; 4) sidechain vector, the relative vector from the node (*C*_α_ atom) to the geometric center of its sidechain atoms; 5) secondary structure class, calculated by DSSP; 6) degree, the number of edges adjacent to the node; 7) eigenvector centrality, a measure of the influence of a node in a connected network.^[^ ^53^ ^]^

For edge settings, three kinds of edge definitions were combined: 1) Distance edges, which exist if the Euclidean distance between two node atoms was less than eight angstroms; 2) k‐NN edges (*k* = 3), which ensure the nearest three neighbor residues of the modification site can involve; 3) Peptide bonds, which connect the sequentially continuous residues within the subgraph. If two nodes meet more than one of these requirements simultaneously, only one edge between them will exist.

### Cross‐Attention Block

Merging the representations of the global and residue‐level evolutionary context and the local structure graph properly was vital for our prediction. Our aim was to predict the functional effect of phosphorylation sites, which can be explained as the effect of a phosphorylation site on its corresponding protein function. Therefore, the merging module was responsible for cross‐talking between the site subgraph and its protein sequence context. Here, the cross‐attention block was used to fuse the two representations. The concept of cross‐attention was from the Perceiver, a variant of transformer architecture.^[^ ^54^ ^]^ Perceiver obtains good performance on multi‐modal inputs to compress dimension and improve the computing speed. Previous studies have transferred the model to compound‐protein interaction prediction, and Perceiver could be used to extract the effect of ligands on their target proteins.^[^ ^55^ ^]^ In this block, the attention is calculated as Equation (1), where the query *q* was the ESM‐2 embedding while the key *k* and value *v* were from the GCN output.

(1) $$attention=softmax\left(\frac{qk^{T}}{\sqrt{C/d}}\right)v$$

*C* was the embedding dimension and was set as 256. A single head was used, so the number of attention heads *d* was one.

### Self‐Attention Block and Classifier

Inspired by Perceiver IO and to further improve the predictive capacity of our model, a self‐attention module was added after the cross‐attention block.^[^ ^56^ ^]^ The self‐attention matrix of the output of the cross‐attention block was calculated, following Equation (1) as well. Finally, a three‐layer full‐connected neural network was used to complete the functional phosphorylation site prediction. The numbers of neurons in each layer were 256, 256, and 512, respectively.

### Model Training and Validation

The deep neural network was built with PyTorch library and trained on NVIDIA Titan Xp GPU.^[^ ^57^ ^]^ DGL and DGL‐LifeSci were used for graph neural networks.^[^ ^58^, ^59^ ^]^ During training and validation, the five‐fold cross‐validation strategy was used. The model was trained on the training set, optimized parameters on the validation set, and evaluated classification performance on the test set. Basic hyperparameter settings are listed in Table S3 (Supporting Information). After trying and comparing different parameter combinations, the batch size 128 and learning rate 1e‐4 were chosen. Epoch was set to 50 for each fold. To avoid overfitting, early stopping was adopted and the dropout rate was set to 0.3. The loss functions of the two prediction tasks were the binary cross entropy loss as well, calculated as the Equation (2), where *y* was the real label of data samples and $\hat{y}$ was the predicted probability. The optimizer was AdamW, which outperforms Adam.^[^ ^60^ ^]^

(2) $$Loss=-\left(ylog\left(\hat{y}\right)+\left(1-y\right)\log\left(1-\hat{y}\right)\right)$$

### Evaluation Metrics

Seven typical classification metrics were used to evaluate the predictive performance of different models. These metrics were the area under the receiver operating characteristic curve (AUROC), the area under the precision‐recall curve (AUPR), accuracy, precision, recall, F1‐score, and Mathew's correlation coefficient (MCC).

(3) $$Accuracy=\frac{TP+TN}{TP+TN+FN+FP}$$

(4) $$Precision=\frac{TP}{TP+FP}$$

(5) $$Recall=\frac{TP}{TP+FN}$$

(6) $$F1-score=\frac{2Precision\times Recall}{Precision+Recall}$$

(7) $$MCC=\frac{TP\times TN-FP\times FN}{\sqrt{\left(TP+FN\right)\left(TN+FP\right)\left(TP+FP\right)\left(TN+FN\right)}}$$

where *TP*, *TN*, *FP*, *FN* were referred to the numbers of true positive, true negative, false positive, and false negative samples, respectively.

## Conflict of Interest

The authors declare no conflict of interest.

## Supporting information



Supporting Information

## Data Availability

The data that support the findings of this study are available in the supplementary material of this article.
