# Supplementary material for: MMFuncPhos: A Multi‐Modal Learning Framework for Identifying Functional Phosphorylation Sites and Their Regulatory Types
Source: Adv Sci (Weinh). 2025 Jan 13;12(9):2410981. doi: 10.1002/advs.202410981 (PMC11884596; doi:10.1002/advs.202410981)
Supplement: Supplementary file 1 — Supporting Information [file ADVS-12-2410981-s001.pdf]

ADVANCED SCIENCE
Open Access

Supporting Information

for *Adv. Sci.*, DOI 10.1002/advs.202410981

MMFuncPhos: A Multi-Modal Learning Framework for Identifying Functional Phosphorylation Sites and Their Regulatory Types

*Juan Xie, Ruihan Dong, Jintao Zhu, Haoyu Lin, Shiwei Wang and Luhua Lai**

Supporting Information

MMFuncPhos: A Multi-Modal Learning Framework for Identifying Functional Phosphorylation Sites and Their Regulatory Types

*Juan Xie, Ruihan Dong, Jintao Zhu, Haoyu Lin, Shiwei Wang, and Luhua Lai**

This file contains:

Table S1. Statistics of Functional Phosphorylation Site Data Set

Table S2. Statistics of Enzyme Activity Regulatory Site Data Set

Table S3. Hyperparameter Settings of the Model

Table S4. Comparisons with Different Parameters

Table S5. Results of the Ablation Study

Table S6. Results of Enzyme Activity Regulatory Types Prediction

Details for knowledge distillation

Figure S1. The performance of the model when using different ratios of positive to negative samples.

Figure S2. Relationship between model predictions and pLDDT scores.

Figure S3. Developing the new predictor through knowledge distillation.

Figure S4. Parameters settings during the distillation of the functional phosphorylation site prediction model.

Figure S5. Parameters settings during the distillation of the regulatory type prediction model.

Table S1. Statistics of Functional Phosphorylation Site Data Set

Set	Train	Test	Total
Functional	5835	1410	7245
Non-functional	5835	1410	7245

Table S2. Statistics of Enzyme Activity Regulatory Site Data Set

	Train	Test	Total
Induced	589	154	743
Inhibited	242	58	300

Table S3. Hyperparameter Settings of the Model

Parameter	Value
Batch size	128
Learning rate	1e-4
Epoch	50
Loss	Binary cross entropy
Optimizer	AdamW
GCN layers	2
Graph radius	8 Å
GCN hidden dimension	64
Node embeddings	35
MLP dimensions	256, 256, 512

Table S4. Comparisons with Different Parameters

Parameter	Value	AUROC	AUPR	Accuracy	Precision	Recall	F1	MCC
Graph radius	6	0.8145	0.8342	0.7546	0.7688	0.7464	0.7575	0.5094
	**8**	0.8311	0.8576	0.7676	0.7849	0.7532	0.7687	0.5359
	10	0.7937	0.8192	0.7291	0.7397	0.7277	0.7336	0.4581
	12	0.8033	0.8187	0.7404	0.7574	0.7262	0.7415	0.4814
Batch size	32	0.8299	0.8375	0.7600	0.7611	0.7752	0.7681	0.5196
	64	0.8218	0.8318	0.7524	0.7613	0.7532	0.7572	0.5046
	**128**	0.8311	0.8576	0.7676	0.7849	0.7532	0.7687	0.5359
	256	0.7930	0.8301	0.7527	0.7897	0.7057	0.7453	0.5095
Learning rate	1e-3	0.7841	0.7871	0.7204	0.7426	0.6957	0.7184	0.4422
	**1e-4**	0.8431	0.8588	0.7956	0.8042	0.7950	0.7996	0.5912
	1e-5	0.8311	0.8576	0.7676	0.7849	0.7532	0.7687	0.5359
Dropout	0.1	0.8151	0.8426	0.7429	0.7534	0.7411	0.7472	0.4858
	0.2	0.8311	0.8576	0.7676	0.7849	0.7532	0.7687	0.5359
	**0.3**	0.8362	0.8603	0.7767	0.7901	0.7688	0.7793	0.5537
GCN layer	1	0.7874	0.7602	0.6989	0.7252	0.6645	0.6936	0.4002
	**2**	0.8311	0.8576	0.7676	0.7849	0.7532	0.7687	0.5359
	3	0.8151	0.8005	0.7516	0.7659	0.7426	0.7541	0.5036

Table S5. Results of the Ablation Study

Model	AUROC	AUPR	Accuracy	Precision	Recall	F1	MCC
w/o AF2	0.8222	0.8260	0.7502	0.7512	0.7667	0.7589	0.4999
w/o ESM-2	0.5975	0.5756	0.5578	0.5893	0.4539	0.5128	0.1238
w/o cross-attention	0.7556	0.7579	0.7025	0.6997	0.7355	0.7172	0.4045
w/o self-attention	0.8400	0.8522	0.7869	**0.8155**	0.7553	0.7842	0.5760
**MMFuncPhos**	**0.8462**	**0.8582**	**0.7975**	0.8084	**0.7929**	**0.8006**	**0.5950**

Table S6. Results of Enzyme Activity Regulatory Types Prediction

Strategy	AUROC	AUPR	Accuracy	Precision	Recall	F1	MCC
Direct training	0.6944	0.8655	0.7075	0.8067	0.7857	0.7961	0.2800
Multi-task training	0.7323	0.9089	0.7264	0.7264	**1.0000**	0.8415	0.0000
Fine-tuning, new classifier	0.8236	0.9362	0.6462	0.9759	0.5260	0.6835	0.4489
Fine-tuning, all	**0.8883**	**0.9652**	**0.8443**	**0.9919**	0.7922	**0.8809**	**0.7000**

Knowledge distillation

To leverage learned knowledge from both structures and sequence embeddings, we regard the trained model as the teacher model and extract its output soft labels to guide the training of student MLP.

$$\mathcal{L} = \gamma\mathcal{L}_{label}(\hat{y}, y) + (1-\gamma)\mathcal{L}_{teacher}(\hat{y}^{\tau}, z^{\tau})$$

The total loss $\mathcal{L}$ is composed of two parts, teacher loss and label loss. Teacher loss $\mathcal{L}_{teacher}$ is calculated via KL divergence with the temperature parameter τ (where $\hat{y}^{\tau} = \frac{exp(\hat{y}_i/\tau)}{\sum_i exp(\hat{y}_i/\tau)}, z^{\tau} = \frac{exp(z_i/\tau)}{\sum_i exp(z_i/\tau)}$, z_i is the soft targets of sample i). Label loss $\mathcal{L}_{label}$ is the cross entropy of student model outputs and true binary labels.

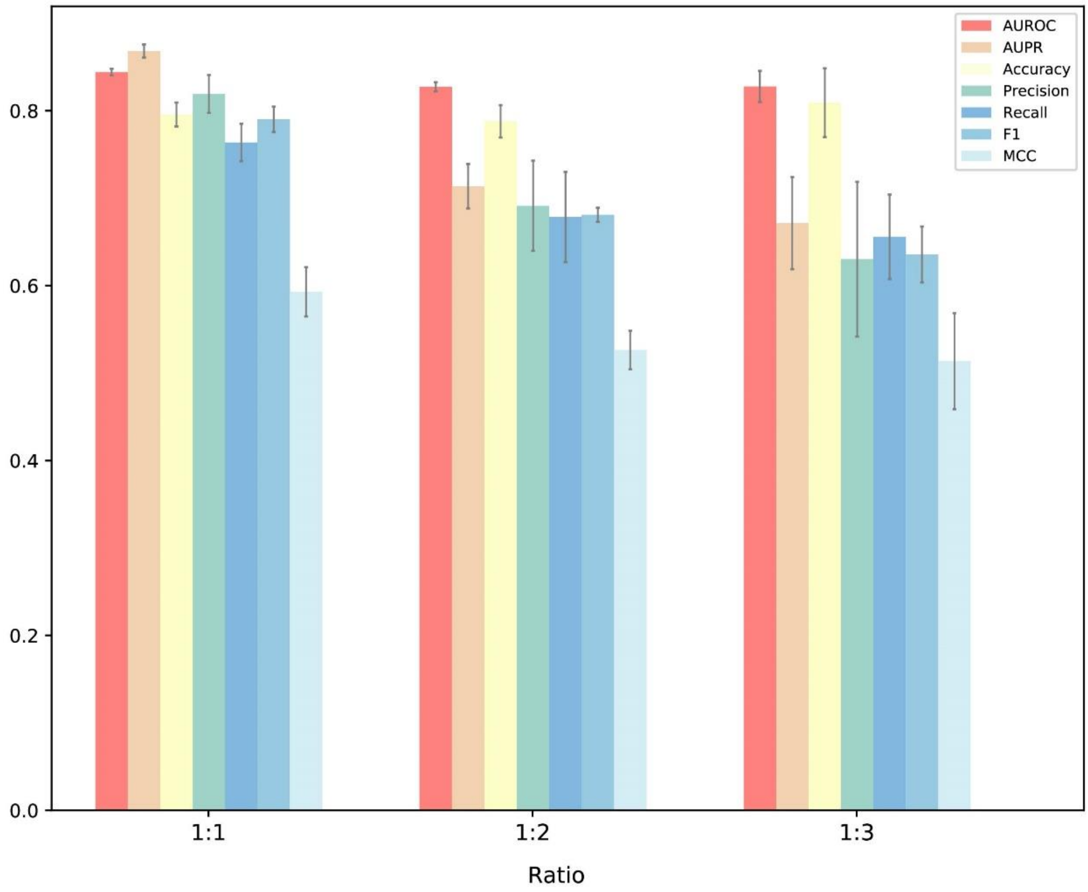

Figure S1. The performance of the model when using different ratios of positive to negative samples. Different ratios of positive to negative samples were considered, such as 1:1, 1:2, and 1:3, and each experiment was repeated three times. All data are expressed as the mean ± standard deviation (SD).

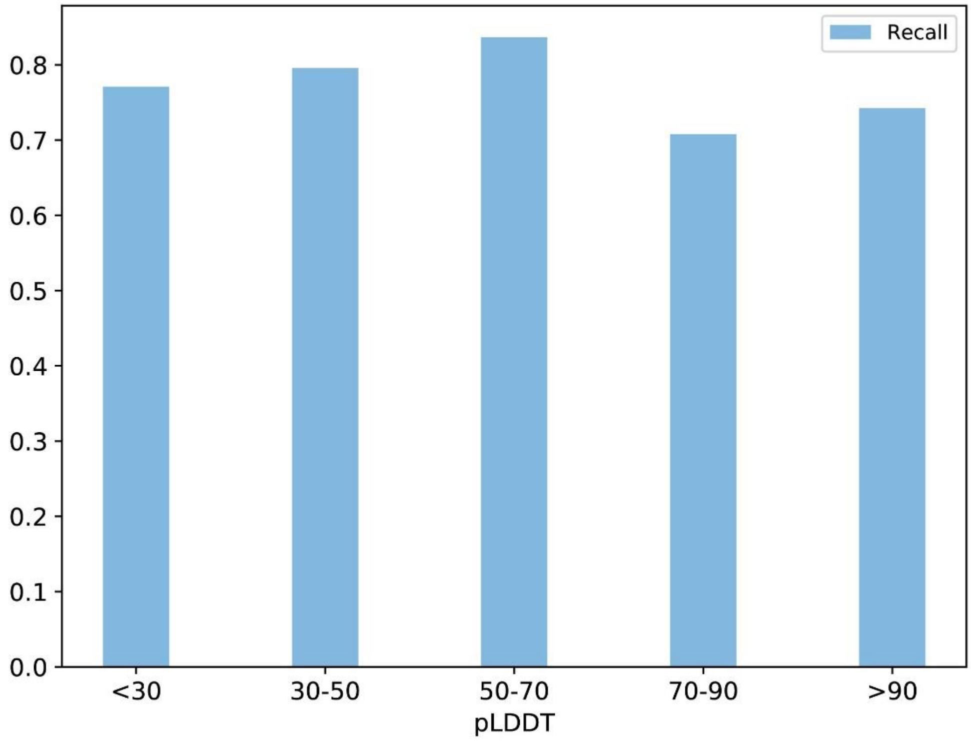

Figure S2. Relationship between model predictions and pLDDT scores. Sites in the test set were divided into different groups according to the pLDDT scores, namely pLDDT > 90, 70 < pLDDT < 90, 50 < pLDDT < 70, 30 < pLDDT < 50, and pLDDT < 30, and then make predictions for each group.

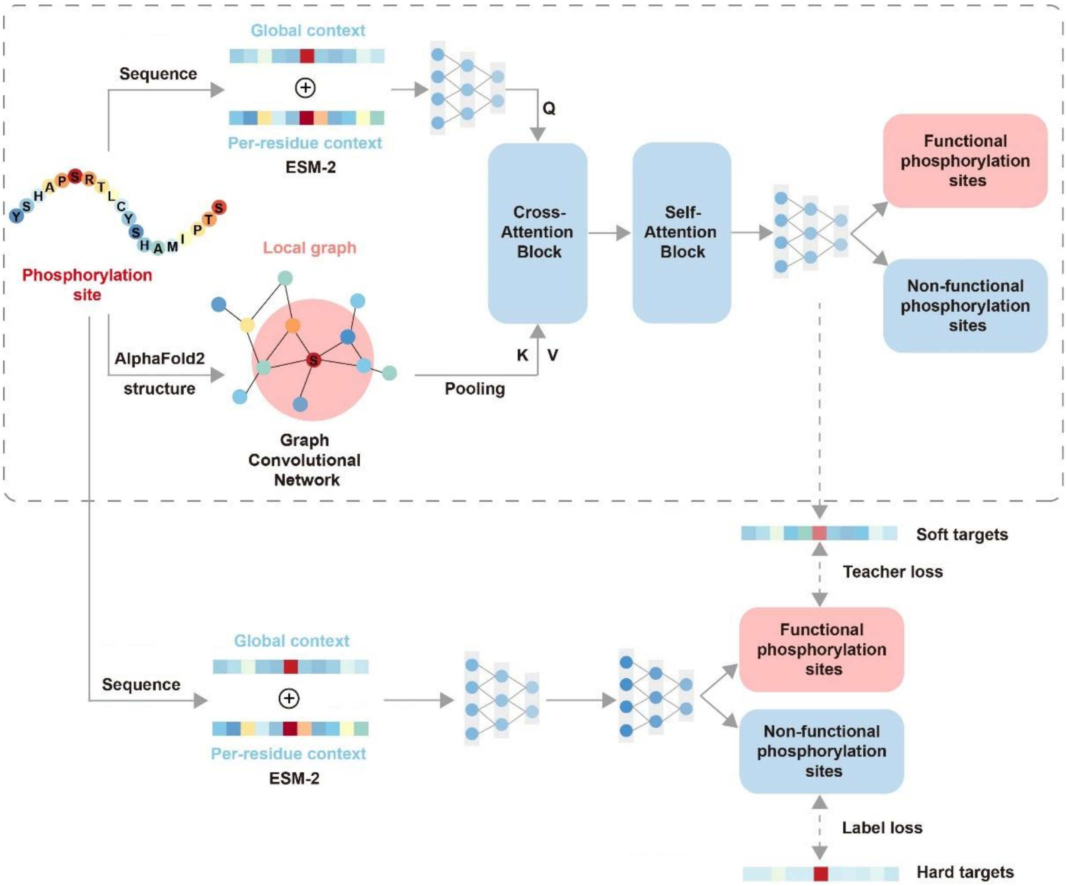

Figure S3. Developing the new predictor through knowledge distillation.

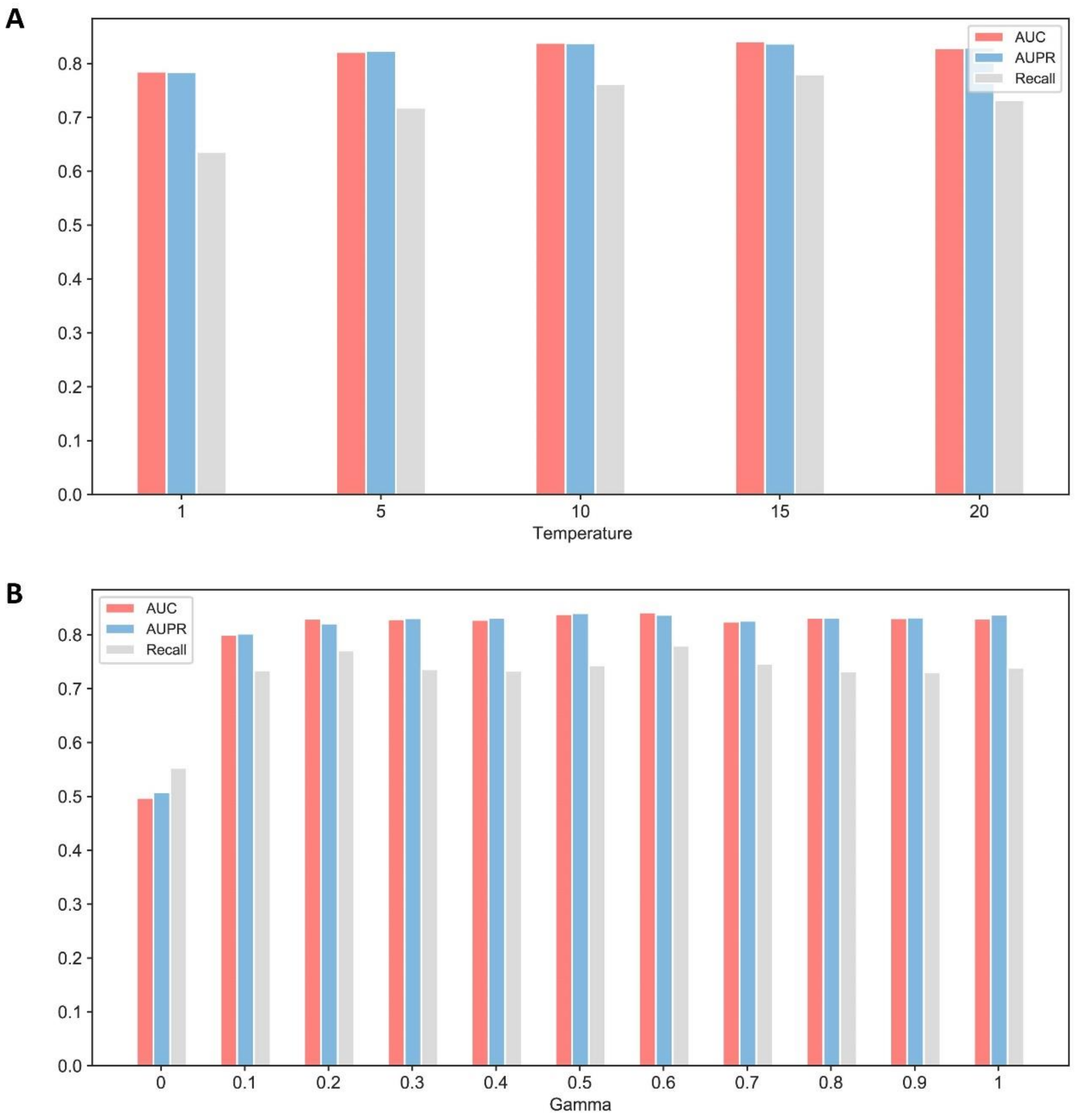

Figure S4. Parameters settings during the distillation of the functional phosphorylation site prediction model. (A) temperature; (B) gamma.

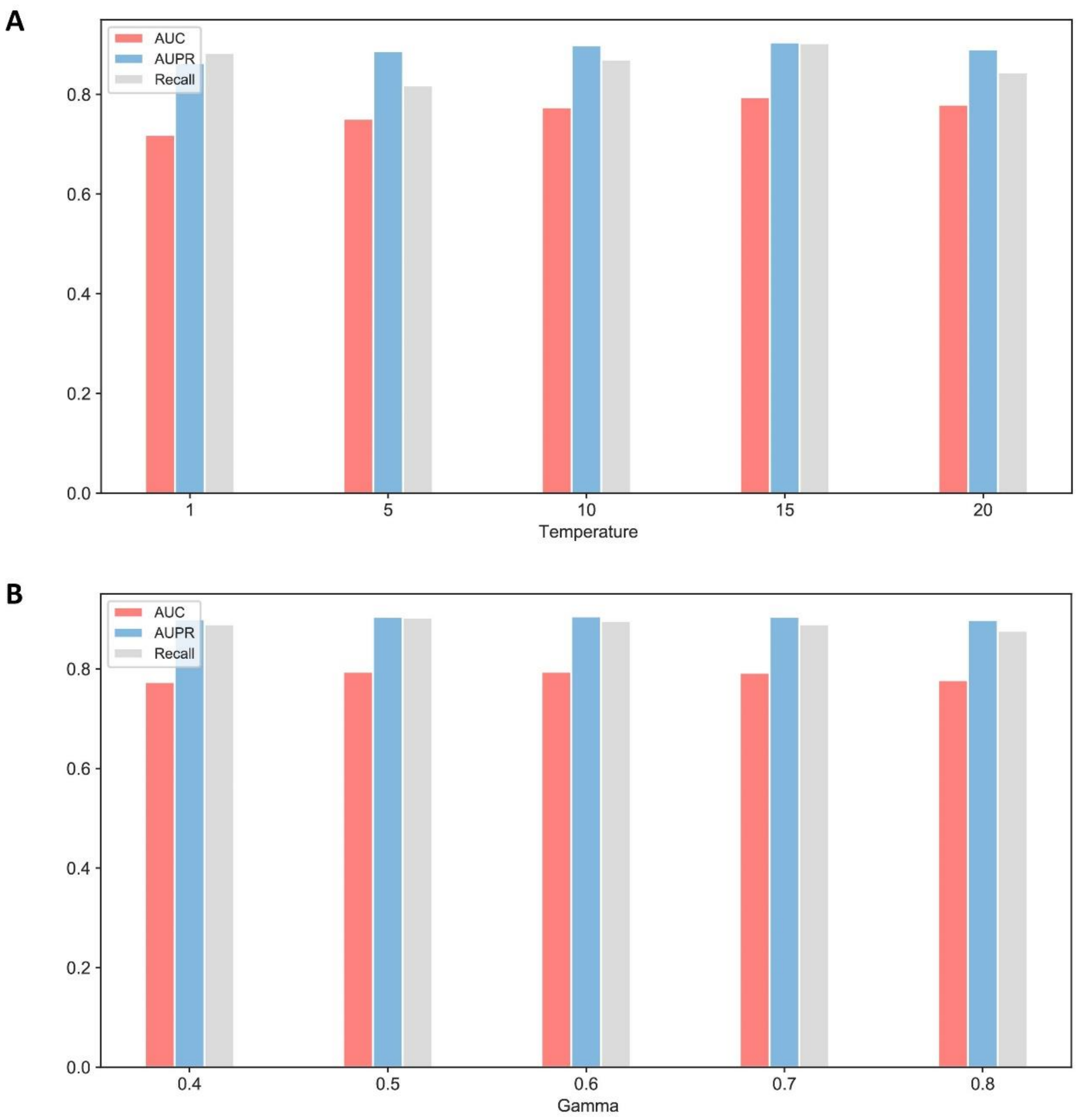

Figure S5. Parameters settings during the distillation of the regulatory type prediction model. (A) temperature; (B) gamma.
